# Prospect of Cascade Catalysis in Magnesium‐Sulfur Batteries from Desolvation to Conversion Reactions

**DOI:** 10.1002/advs.70008

**Published:** 2025-05-28

**Authors:** Qinghua Guan, Jing Zhang, Yongzheng Zhang, Xiaomin Cheng, Jing Dong, Lujie Jia, Hongzhen Lin, Jian Wang

**Affiliations:** ^1^ School of Nano‐Tech and Nano‐Bionics University of Science and Technology of China Hefei 230026 China; ^2^ *i*‐Lab & CAS Key Laboratory of Nanophotonic Materials and Devices Suzhou Institute of Nano‐Tech and Nano‐Bionics Chinese Academy of Sciences Suzhou 215123 China; ^3^ Helmholtz Institute Ulm (HIU) D89081 Ulm Germany; ^4^ Karlsruhe Institute of Technology (KIT) D76021 Karlsruhe Germany; ^5^ School of Materials Science and Engineering Xi'an University of Technology Xi'an 710048 China; ^6^ State Key Laboratory of Chemical Engineering East China University of Science and Technology Shanghai 200237 China

**Keywords:** electrochemical barrier, interfacial desolvation, magnesium‐sulfur battery, sulfur/electrolyte interface, sulfur/polysulfide conversion

## Abstract

Magnesium‐sulfur (Mg‐S) batteries have the advantages of high volumetric energy density, intrinsic safety, and low cost of anode and cathode materials. However, current obstacles that preventing practical applications of Mg‐S batteries are reflected in the sluggish reaction kinetics of insulative sulfur cathode, designs of compatible electrolytes, and surface optimization of Mg anode against passivation. Regarding the sulfur cathodes, the inherent low conductivity, high volumetric changes, and polysulfide shuttling always result in depressive capacity and utilization. As known, the Mg^2+^ carriers are coordinated with solvents in the electrolyte and need to be desolvated before or during the Mg^2+^ participating in the electrochemical reactions. The desolvation steps and the cathodic or anodic redox steps are intercoupled at the electrode/electrolyte interface, which can be regarded as cascade reactions of different pathways. In this review, the efforts to deal with the high‐energy‐barrier processes including Mg^2+^ desolvation, Mg^2+^ migration at the interface and in cathode interior, and sulfur conversions are summarized. Importantly, the possible coupling manners between the above processes are highlighted. Then cascade catalysis strategy for accelerating the desolvation and sulfur conversion kinetics on the premise of superior conductivity is further reviewed along with a variety of characterizations from experiments to theoretical simulations. Finally, future development trends and deep understanding in Mg‐S batteries are prospected.

## Graphical abstract

The dissolution and conversion reactions at the cathode interface of Mg‐S batteries are systematically summarized. This review focuses on the problems and solutions of diffusion/desolvation Mg^2+^ migration barrier at the sulfur/electrolyte interface as well as the current development and design of S cathodes in Mg‐S batteries under catalysis. Consequently, the future development trends and in‐depth understanding of Mg‐S battery are prospected.

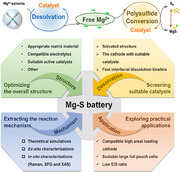

## 1 Introduction

The excessive consumption of traditional energy, such as oil and natural gas, leads to increasingly serious environmental pollution and energy crisis.^[^ ^1^ ^]^ It raises urgent demands to develop environmentally friendly, large‐scale and efficient energy storage devices to continuously store intermittent energy such as wind and solar energy. Among various technologies, secondary batteries are the most promising because of their high energy conversion efficiency, long service life and potential low cost.^[^ ^2^ ^]^ In the last decade, a variety of beyond Li batteries have been investigated, including alkali metal‐sulfur batteries, such as room‐temperature (RT) sodium‐sulfur (Na‐S) and potassium‐sulfur (K‐S) batteries, and other metal‐sulfur batteries, such as magnesium‐sulfur (Mg‐S), aluminum‐sulfur (Al‐S), zinc‐sulfur (Zn‐S), and calcium‐sulfur (Ca‐S) batteries. However, emerging metal‐sulfur batteries face rigorous and complex challenges that must be addressed before they can be implemented in practical cells. Compared with lithium ion batteries (LIBs), magnesium ion batteries (MIBs) can provide more electrons per unit volume in nature, making it output two‐time higher volume capacity than that of LIBs.^[^ ^3^ ^]^ Meanwhile, magnesium has rich resources and environmental friendliness in the earth.^[^ ^4^ ^]^ Since 2000, substantial progresses have been made on developing magnesium batteries.^[^ ^5^ ^]^ However, there remain some key problems that seriously hinder the commercialization of rechargeable magnesium batteries (RMBs).^[^ ^6^ ^]^ Particularly, the strong polarization effect and slow diffusion kinetics of the high charge‐density Mg^2+^ bring great challenges to the development of cathode materials.^[^ ^7^ ^]^

Among the various types of magnesium batteries, Mg‐S batteries showcase promising rechargeable battery chemistries in advantages of: (1) a high theoretical specific capacity (1672 mA h g^−1^), making it an attractive conversion cathode;^[^ ^8^ ^]^ (2) high theoretical energy densities of 1684 W h kg^−1^ (gravimetric) and 3221 W h L^−1^ (volumetric) with suitable working potential;^[^ ^9^ ^]^ (3) elemental sulfur being abundant on the earth and suitable for mass production. Despite the great promise of Mg‐S batteries, there are still many scientific and technical issues that need to be addressed.^[^ ^6^, ^10^ ^]^ The problems mainly focus on that: (1) the active material (sulfur) is an electronic and ionic insulator, which needs conductive network matrix to load sulfur with high surface area;^[^ ^11^ ^]^ (2) soluble polysulfides tend to form and shuttle to Mg anode, deteriorating metallic Mg surface;^[^ ^11^, ^12^ ^]^ (3) large volume changes occurs and destroys the matrix structure, resulting in low sulfur utilization;^[^ ^13^ ^]^ (4) “solid‐liquid‐solid” phase transformation with sluggish ion transport leads to depressive reaction kinetics.^[^ ^14^ ^]^ In short words, the Mg‐S batteries system faces serious problems of rapid capacity degradation, poor efficiency, and large over‐potential, which possibly can be ascribed to the detrimental effect of the dissolved polysulfides on the properties of the electrolytes and the sluggish electrochemical reactivity of insoluble MgS_2_/MgS species.^[^ ^7^, ^15^ ^]^

**Figure 1:**
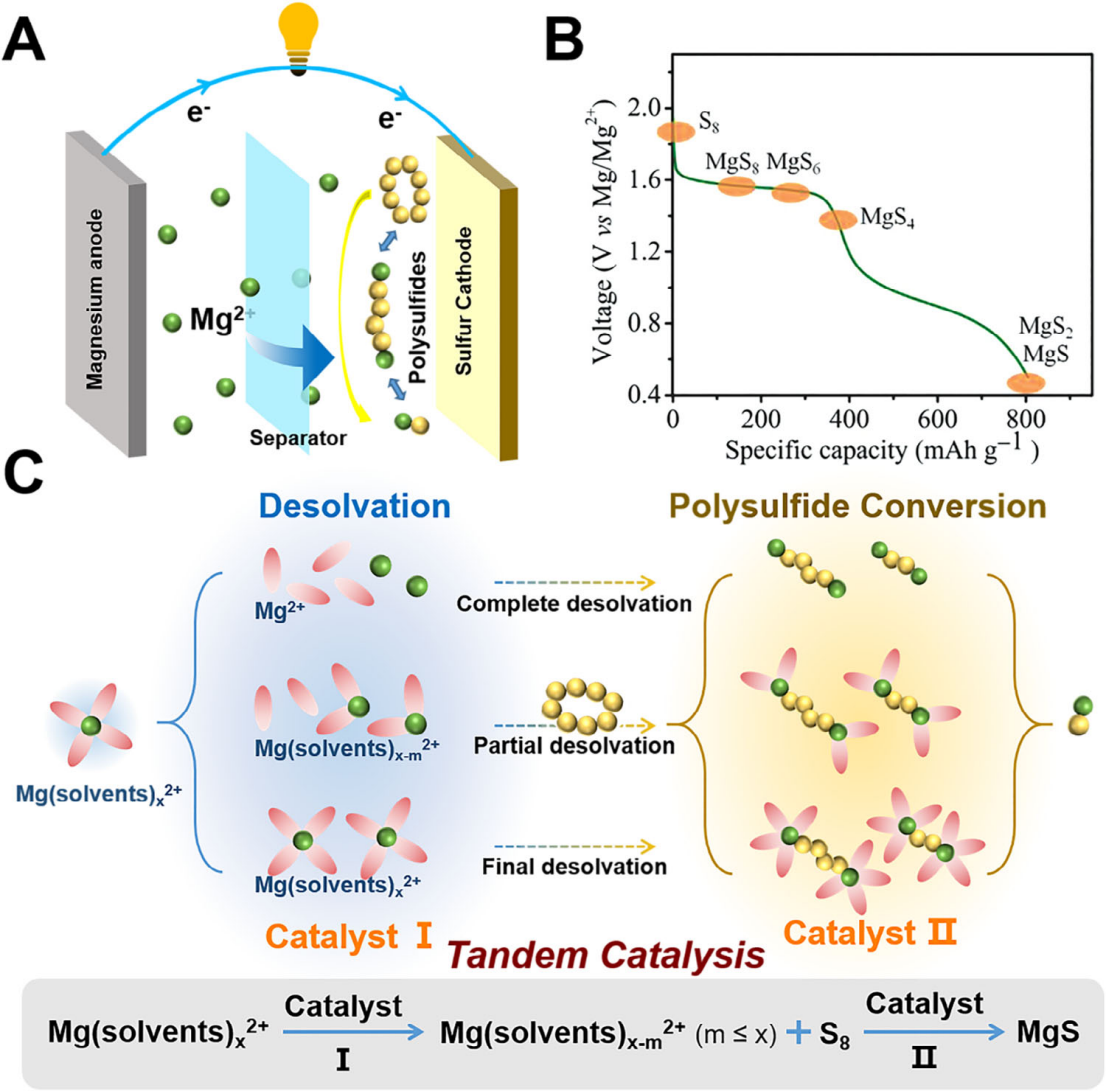
A) Schematic diagram of the Mg‐S battery. B) The proposed discharge profile of Mg‐S battery. Reproduced with permission.^[^ ^7b^ ^]^ Copyright 2014, WILEY‐VCH. C) Schematic illustration of possible desolvation processes and typical tandem catalytic reactions based on the most common reaction pathways on sulfur cathode.

Generally, the Mg‐S battery consists of magnesium metal anode, sulfur‐based cathode, and compatible electrolyte, which is similar to Li‐S battery.^[^ ^16^ ^]^ During the discharge process, the Mg metal on the anode side is oxidized to form Mg^2+^, and electrons flow from the anode through an external circuit to the sulfur cathode, where the sulfur reacts with the Mg^2+^ and electrons to form MgS (**Figure** **1A**):

(1) $$\mathrm{Magnesium}\;\;\mathrm{anode}\;\left(-\right):\;\mathrm{Mg}\;\to \;Mg^{2+}+2e^{-}$$

(2) $$\mathrm{Sulfur}\;\;\mathrm{cathode}\;\left(+\right):\;S_{8}+8Mg^{2+}+16e^{-}\to \;8\mathrm{MgS}$$

However, the reduction of sulfur during the actual discharge process of Mg‐S batteries is very complex and involves the phase transition of various sulfides, which is still unclear (Figure 1B). In 2015, Zhao Karger et al. first proposed a three‐step reduction mechanism of sulfur based on electrochemical characterization and *ex‐situ* X‐ray photoelectron spectroscopy (XPS).^[^ ^17^ ^]^ The first step is assigned to a solid‐liquid two‐phase reduction of sulfur to MgS_8_, which subsequently dissolves into the electrolyte, and following reduction from high‐order MgS_8_ to low‐order polysulfides (MgS_4_) occurs in the liquid phase. In subsequent steps, the dissolved low‐order polysulfide (MgS_4_) is reduced to MgS_2(solid)_. The last reduction step (MgS_2_‐MgS), which occurs in the solid state, suffers from high kinetic barriers and high polarization. In the subsequent work, the researchers conducted a more in‐depth study of the mechanism of Mg‐S batteries with more testing methods,^[^ ^18^ ^]^ such as *in‐situ* X‐ray diffraction (XRD), X‐ray absorption near edge structure (XANES), resonant inelastic X‐ray scattering (RIXS), etc., and found that the specific polysulfides conversion in different electrolytic liquid systems was different.^[^ ^7c^ ^]^ In the Mg(TFSI)_2_ system, the reduction process of sulfur is mainly divided into three steps: when the voltage range is 2.4‐1.5V, the solid phase of sulfur is first converted into long chain MgS_8_, completing the transformation from solid phase to liquid phase; At the voltage platform of 1.5V, the long chain MgS_8(solution)_ is quickly converted to the short chain MgS_2(solid)_. Finally, MgS_2_ is converted to MgS at 1.5‐0.5V, and since this step occurs in solid phase, it has a large reaction barrier, low utilization rate of sulfur, and poor reversibility of the battery.^[^ ^19^ ^]^ In Mg(HMDS)_2_‐AlCl_3_, the transformation process of S→MgS_8_ /MgS_4_→Mg_3_S_8_→MgS exists.^[^ ^20^ ^]^ In the solid phase, magnetization slows down the conversion of long‐chain polysulfides to MgS. The nature of polysulfides like Mg_3_S_8_, MgS_2_, or MgS is insulating, alongside the lower diffusivity of Mg^2+^ within solid phase, leading to massive overpotential on the last step of discharge likewise final products re‐conversion to long‐chain polysulfide in charging process is lacking.

As extensive studies have been focused on monitoring the sulfur species, little attention has been paid to the behaviors of Mg^2+^ in the practical reactions. Due to the high charge density of the divalent ion, the Mg^2+^ and solvents will form a large solvation structure and generate clusters with huge steric effect, which not only hinders the migration of Mg^2+^ in the electrolyte, but also results in successively slow reaction kinetics.^[^ ^21^ ^]^ A requisite for the redox transformation reactions to proceed smoothly and efficiently at the conversion‐type electrodes is the fast dissociation of the solvated Mg(solvent)_x_^2+^ complex, which is often neglected in the physical models described in the existing literature.^[^ ^22^ ^]^ We propose three possible desolvation processes based on the most common reaction pathways (Figure 1C): 1) The Mg(solvents)_x_^2+^ structure first dissolves to form free Mg^2+^, and then combines with sulfur to participate in the polysulfides transformation process; 2) The Mg(solvents)_x_^2+^ structure is partially dissolved to form Mg(solvents)_x‐m_^2+^ (m < x) before it is combined with sulfur, and Mg(solvents)_x‐m_^2+^ (m < x) participates in the polysulfides conversion process in the liquid phase, then the remaining solvent structure is removed again before it is finally converted into solid MgS; 3) The Mg(solvents)_x_^2+^ structure retains its original form when combined with sulfur, participates in the polysulfur conversion process in the liquid phase, and undergoes desolvation when finally converted to solid MgS. Although it is still unclear exactly at which stage of the reaction the desolvation of magnesium ions takes place, the initial and final states of the reaction are consistent and determined, that is from solid sulfur to liquid polysulfides and finally converted into solid MgS, then regardless of the carrier form of the intermediate process, desolvation must occur in the whole process of the reaction. In particular, the MgS in the final state is solid, so the magnesium ion in it must be in a free state, so desolvation is inevitable.

In this review, the strategy of tandem catalysis is proposed to accelerate the reactions in Mg‐S batteries in dealing with severe solvation behaviors and sluggish conversion reactions of sulfur. Specifically, as shown in Figure 1C, the free Mg^2+^ is both the product from the desolvation reaction and also serve as the reactant of the sulfur/sulfide redox reactions, showing the tandem reactions with barriers. To decrease the related barriers with enhanced redox kinetics, the catalysts are usually introduced to effectively accelerate the dissociation of Mg(solvents)_x_^2+^ and release sufficient free Mg^2+^ at the cathode/electrolyte interface. Meanwhile, in the redox reactions of polysulfides and sulfur, the series reactions include polysulfide interconversions and MgS oxidation will be propelled under the help of catalysis in the sulfur interior. Initially, the catalytic effects of dissociating Mg^2+^ with decreased desolvation barrier at the cathode/electrolyte interface are analyzed discussed; and then, the kinetics‐enhanced existence of sulfur conversion processes is summarized from matrix conductivity, adsorption, to electrochemical redox kinetics of sulfur species with the deep understanding from experiment to theory. Finally, the key issues preventing the development of Mg‐S batteries are prospected in molecular level and practical application.

## 2 Fast Desolvation for Rapid Mg^2+^ Migration at the Sulfur/Electrolyte Interface

As discussed above, in the actual battery environment, before reaching the sulfur cathode, Mg^2+^ tends to cooperate with solvent molecules in electrolyte to form Mg(solvents)_x_^2+^. At the sulfur/electrolyte interface, the large size of Mg(solvents)_x_^2+^ cluster must firstly be dissociated to form Mg^2+^, which is the premise of successive polysulfide redox conversion reactions.^[^ ^23^ ^]^ Therefore, the desolvation and diffusion behaviors of Mg^2+^ at the interface or inside the cathode is closely related to the state of the Mg(solvents)_x_^2+^ solvated structure.^[^ ^22^ ^]^ To dissociate the coordinated solvent molecules from the Mg(solvents)_x_^2+^ solvation sheath seems much important to form a free separation Mg^2+^. Generally speaking, the direct separation of solvent molecules from the Mg(solvents)_x_^2+^ complex involves the breaking of the associated chemical coordination bonds, presenting high energy barriers. Therefore, it is a necessity to decrease the interfacial barriers for forming bare Mg^2+^ to diffuse smoothly across the cathode/electrolyte interface.^[^ ^24^ ^]^ In this section, the kinetics of desolvation and diffusion of Mg^2+^ accelerated by catalyst will be discussed.

### 2.1 Atomic Catalysts for Fast Mg^2+^ Desolvation at the Sulfur/Electrolyte Interface

**Figure 2:**
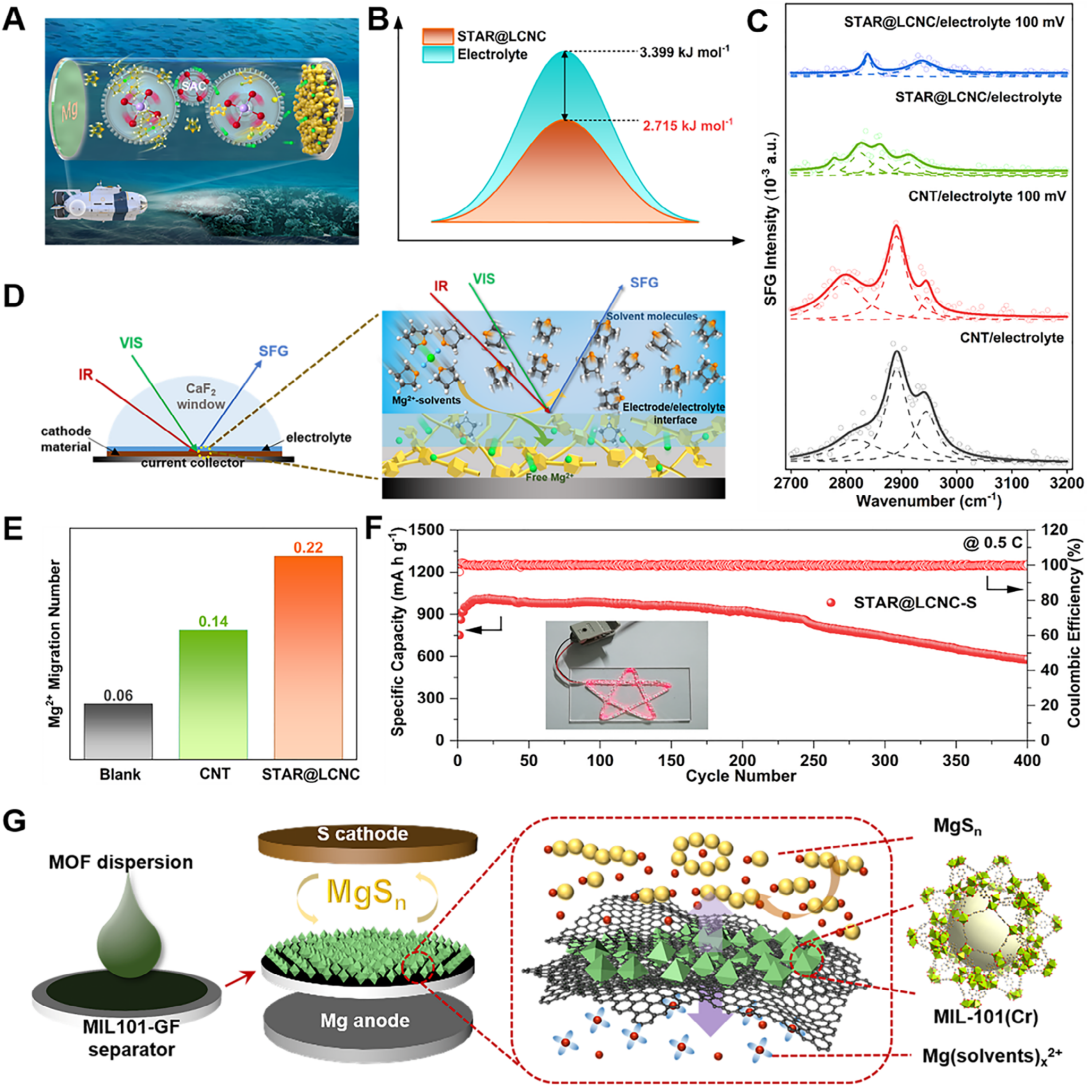
A) The schematic illustrations of self‐tandem catalysis. B) Desolvation barrier of MgCl_2_(THF)_2_ into MgCl_2_·THF and THF on STAR@LCNC or in liquid electrolyte, respectively. C) Schematic illustration of the *in‐situ* SFG probing the electrolyte/catalyst interface and the molecular states of the Mg^2+^ solvation structure in the STAR@LCNC electrode/electrolyte interface after turning bias voltage on. D) The SFG spectra of different adsorption states of Mg^2+^ solvation structure in MgCl_2_‐LiCl/THF electrolyte. E) Magnesium ion migration number for blank magnesium foil, CNT, and STAR@LCNC materials. F) Cycling performance of the STAR@LCNC‐S electrodes for 400 cycles at 0.5 C and the high‐loading cell with STAR@LCNC‐S electrode powers a series of the star‐shaped lights. Reproduced with permission.^[^ ^22^ ^]^ Copyright 2024, The Royal Society of Chemistry. G) Schematic diagram of preparation and structure of MIL101‐GF. Reproduced with permission.^[^ ^33^ ^]^ Copyright 2025, Wiley‐VCH GmbH.

It is well known that accelerating the transport of ions helps to enhance the reaction kinetics, which in turn effectively improves the battery performance, however, the chemical states of Mg^2+^ present in the battery is seldom discussed at the interface.^[^ ^25^ ^]^ Existing studies suggest that the state of carriers such as Li^+^ and Mg^2+^ depends on the continuous reaction.^[^ ^23c^ ^]^ In a common electrolyte, Mg^2+^ together with solvents will form a large solvation shell structure and produces huge steric effect, thus leading to slow desolvation kinetics.^[^ ^26^ ^]^ For the first time, we had proposed a self‐tandem catalyst for rapid Mg^2+^ desolvation and sulfur conversion to controllably solve the problem of sluggish kinetics.^[^ ^22^ ^]^ We selected lithium‐containing electrolyte and copper current collector. Li‐containing electrolytes are widely used in magnesium battery systems,^[^ ^27^ ^]^ where the introduction of Li^+^ effectively addresses the deficiencies of single magnesium salt electrolytes in terms of ionic conductivity, oxidation stability, and interface compatibility. In organic solvents, LiCl always accelerates the dissolution of MgCl_2_, facilitating the removal of the passivating MgCl_2_ layer on the Mg anode and activating the electrolyte/anode interface. Meanwhile, the incorporation of Li^+^ also alters the solvation structure with decreased barrier, leading to a notable enhancement in the desolvation kinetics of Mg^2+^.^[^ ^28^ ^]^ However, the reduction potential of Li^+^ (≈3.04 V vs. SHE) is significantly lower than that of Mg^2+^, which also prevents the possibility of Mg‐Li co‐deposition.^[^ ^10^, ^29^ ^]^ Regarding the current collector, previous studies have shown that Cu current collector can significantly improve the electrochemical performance of Mg‐S batteries.^[^ ^30^ ^]^ The interaction between copper and sulfur, mediated by copper‐sulfur compounds, partially suppresses the dissolution of polysulfides in the electrolyte. Flower‐like CuS will form on the surface during the preparation process of electrodes using copper current collectors, altering the reaction path of elemental sulfur during charge/discharge processes. The CuS in the cathode is converted to Cu_2_S during the initial discharge process, and then Cu_2_S is transformed back to Cu, while the S in the electrode is converted to MgS through MgS_x_. During the charging process, Cu can react with MgS to form Cu_2_S, and then partially convert to S through MgS_x_, decreasing the barriers. However, when the copper current collector is above 1.8 V (vs. Mg/Mg^2+^), electrochemical corrosion will occur, which will cause irreversible consumption of the cathode current collector and the Mg metal anode, ultimately leading to battery failure.^[^ ^31^ ^]^ As shown in **Figure** **2A**, the strategy of atomic zinc catalyst at the electrolyte/electrode interface was effectively designed to promote the dissociation of the solvated structure, releasing the more free Mg^2+^ with the lower desolvation energy barriers. Theoretical simulations were performed to understand the decomposition behaviors of MgCl(THF)*_x_* ^+^ into MgCl_2_·THF and THF molecules on STAR@LCNC. Obviously, the corresponding dissociation energy barrier of ≈2.7 kJ mol^−1^ is achieved under the help of atomic catalyst, which is lower than that in the electrolyte (MgCl_2_‐LiCl/THF) (Figure 2B). Usually, the interfacial arrangement of cations and anions can be described by the double‐layer model with the inner Helmholtz layer and the outer diffuse layer.^[^ ^32^ ^]^ As one would expect that the charged species in the diffuse layer are dominated by solvated ions, the ions in the inner layer can exist as solvated, partially solvated, or free ones without any solvent shell, depending on the properties and local environments of electrode surface. As illustrated in Figure 2C, the *in‐situ* interface‐sensitive sum frequency generation (SFG) spectroscopy was constructed to monitor the desolvation behavior evolution of the solvated Mg^2+^. Characteristic C‐H stretching vibrations of the solvent (THF) were investigated in the 2700–3000 cm^−1^ range at the electrode/electrolyte interface with *ssp* polarizations (Figure 2D). Two groups are observed, namely, one group (2826 and 2860 cm^−1^) assigned to pure THF and the other group (2778 and 2916 cm^−1^) corresponded to solvated THF in the solvation shell. At the STAR@LCNC/electrolyte interface, the SFG signal of the solvated shell THF almost disappears in comparison with that on CNT surface when a bias voltage of 100 mV is applied, whereas the other peaks located at 2840 and 2938 cm^−1^ were assigned to free, nonspecifically adsorbed THF molecules. Meanwhile, the fast desolvation behaviors are also reflected in the increased ion kinetics. The improvement of the Mg^2+^ transference number from 0.14 for CNT to 0.22 for STAR@LCNC also suggests that STAR@LCNC significantly improves ion diffusion and migration kinetics by promoting Mg^2+^‐solvent dissociation (Figure 2E). Consequently, the as‐fabricated STAR@LCNC‐S cathodes show the best ever rate performances up to 2 C and with the so‐far longest lifespan of 400 cycles. In addition, by increasing the mass loading to 4 mg cm^−2^, the cell can stabilize the areal capacity of 2.92 mA h cm^−2^ and power a series of star lamps (Figure 2F). This catalytic strategy opens up new routes to design and fabricate highly active catalysts with favorable interface environments with fast desolvation kinetics to develop faster, longer‐lasting, higher‐energy density Mg‐S batteries. As shown in Figure 2G, a catalytic strategy of combining porous sieve desolvation and molecular electrocatalysis was proposed to dissociate Mg(solvents)_x_^2+^ structure by using MIL‐101(Cr), a representative metal‐organic frameworks (MOF) material, as an interfacial promoter to achieve fast Mg^2+^ desolvation and sulfur conversions without affecting the performances of the electrolyte in liquid phase.^[^ ^33^ ^]^

### 2.2 Promoting Mg^2+^ Migration at the Sulfur/Electrolyte Interface

**Figure 3:**
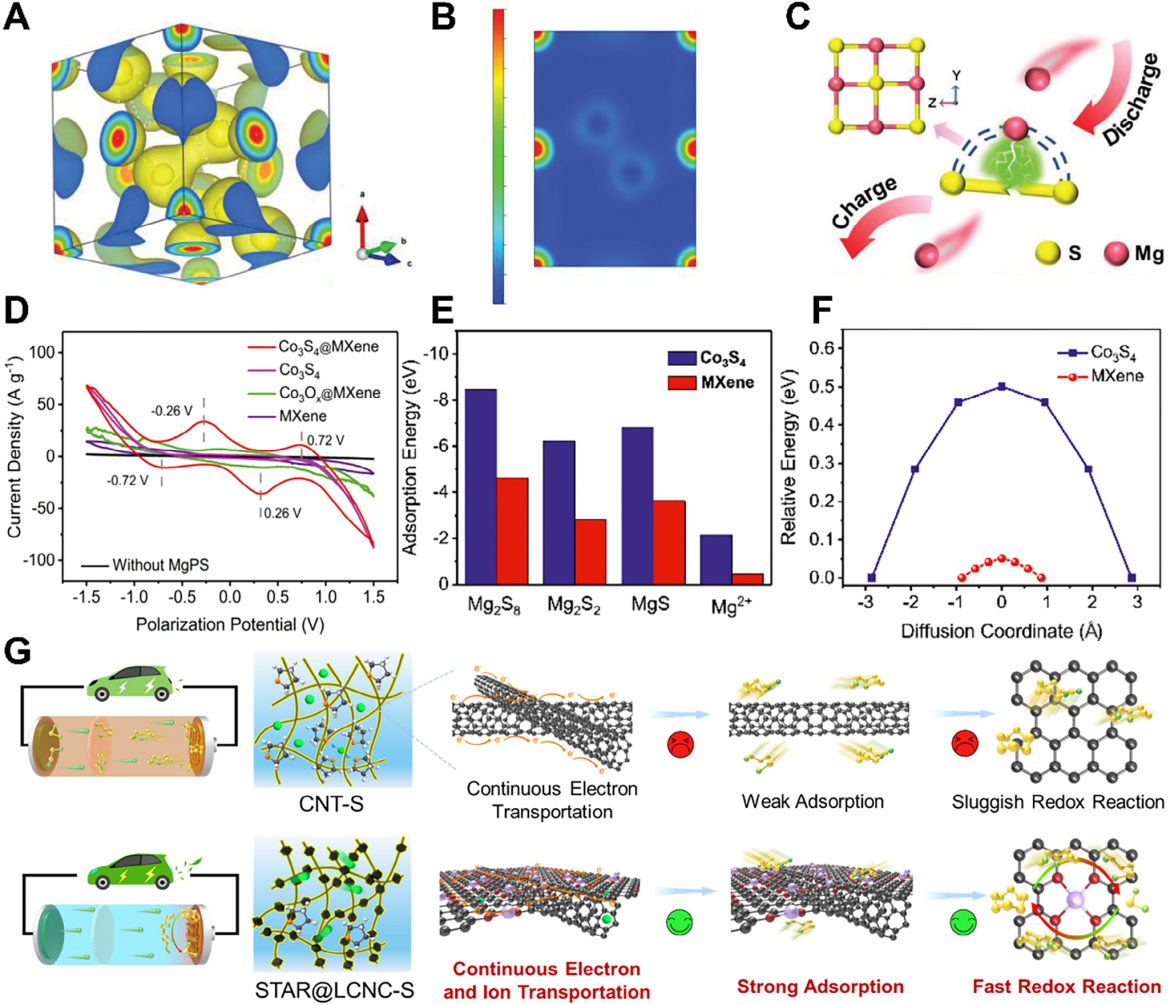
A) 3D charge density map. B) 2D charge density map. C) Reaction mechanism during discharge. Reproduced with permission.^[60]^ Copyright 2022, Wiley‐VCH GmbH. D) Comparisons of the CV profiles of Co_3_S_4_@MXene, Co_3_S_4_, CoO_x_@MXene, and MXene as host materials for the magnesium polysulfides conversion. E) Adsorption energies of the magnesium polysulfides and Mg ion. F) Energy profiles for diffusion processes of Mg ion on the Co_3_S_4_ and MXene, respectively. Reproduced with permission.^[^ ^25b^ ^]^ Copyright 2020, Elsevier. G) The proposed catalytic mechanism without atomic reactors in CNT‐S or with STAR@LCNC‐S in Mg‐S batteries. Reproduced with permission.^[^ ^22^ ^]^ Copyright 2024, The Royal Society of Chemistry.

The strong electrostatic interaction between the bivalent Mg^2+^ and host materials leads to the slow diffusion kinetics of Mg^2+^ and poor cycling stability in the process of Mg^2+^ insertion/extraction. How to promote the cathode reaction kinetics and realize the rapid conduction diffusion of Mg^2+^ plays a crucial role in the reversible Mg‐S batteries. For example, Ye et al. studied an anion‐rich sulfide electrode with carbon nanotubes (NiS_2_/NCNT) as the cathode material.^[^ ^25a^ ^]^ The NiS_2_/NCNT with good Mg^2+^ storage performance was synthesized by simple *in‐situ* growth of NiS_2_ nanoparticles on NCNT. NiS_2_ with large regular cavity structure and abundant sulfur‐sulfur (S‐S) bonds had high electronegativity, and can provide a large number of active sites and unobstructed transport paths for insertion‐disinsertion of Mg^2+^. Charge density analysis shows that Mg^2+^ with positive bivalent charge enters adsorbs near the S‐S bond with concentrated negative charge (**Figure** **3A–B**). As the S‐S bond breaks, Mg^2+^ forms weakly coordinated Mg‐S bonds with the generated S^2−^ to generate MgS. Since the NiS_2_ crystal structure is porous enough during discharge, Mg^2+^ can pass through freely (Figure 3C).

Accelerating the transport of Mg^2+^ and increasing the conversion rate of polysulfide can effectively improve the electrochemical performance of magnesium sulfur batteries. The heterogeneous structure of Co_3_S_4_@MXene was used as an effective sulfur host for reversible magnesium sulfur batteries.^[^ ^25b^ ^]^ Two pairs of high visible reduction and oxidation peaks, rather than elongated peaks, indicates that it had a good catalytic effect (Figure 3D). As shown in Figure 3E–F, density functional theory (DFT) calculation results showed that the adsorption capability of Co_3_S_4_ for MgPS is much higher than that of MXene matrix, which indicating that Co_3_S_4_ is an efficient anchoring site for MgPS on conductive MXene matrix. The adsorption capacity of MXene matrix for Mg^2+^ was lower than that of Co_3_S_4_, indicating that MXene matrix could effectively accelerate the diffusion kinetics of magnesium ions. In conclusion, Co_3_S_4_ could catalyze the transformation of MgPS by chemical anchoring, while MXene provided smooth diffusion and transfer of magnesium ions for the rapid reaction dynamics of Mg‐S batteries. The serially‐assembled train‐like atomic reactors of zinc atomic catalysts employed onto long‐conductive nitrogen‐doped nanocarbons are designed (STAR@LCNC), serving as kinetics promoter in Mg‐S batteries.^[^ ^22^ ^]^ The STAR on the cross‐linked electronic network guarantees high catalytic activity towards rapid Mg^2+^ transport in the cathode interior and exchange at the electrode/electrolyte interface, and the bead‐shape (pearl) domains provide the triphasic local environment to allow efficient utilization of SACs to promote the electrochemical kinetics of sulfur reduction reactions (SRRs) and sulfide oxidation reactions (SORs). Compared with the pristine CNT‐S cell with severe shuttling effect (Figure 3G), the STAR@LCNC modified Mg‐S cell exhibits the continuous electron and ion transportation, strong adsorption toward MgS_x_, and fast SRRs and SORs by introducing STAR promoters. These synergies guaranteed the high reversible electrochemical reaction and high sulfur utilization rate of magnesium sulfur batteries.

**Figure 4:**
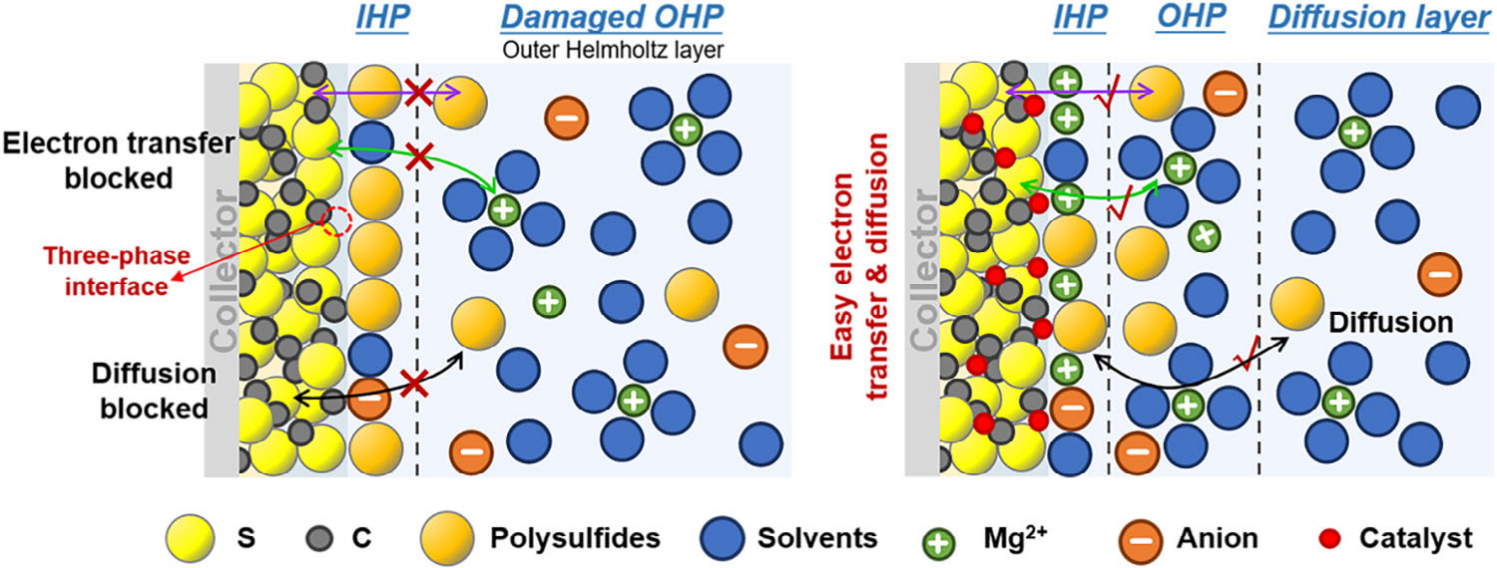
Schematic illustration of the EDL structure at the cathode/electrolyte interface without/with catalyst.

Mg‐S chemistries are most interfacial reactions occurring at the interface between the conductive matrix and the electrolyte. In order to enhance the utilization ratio of sulfur, the overlapped three‐phase interface (conductive carbon matrix/electrolyte /sulfur) should be extended, and appropriate catalytic sites should be introduced at the three‐phase interface to accelerate the combination of Mg^2+^ with S for subsequent sulfur/sulfide conversions. At the same time, it should be noted that an appropriate carbon structure or catalytic site should be designed to limit the diffusion of polysulfides and prevent them from shuttling to the magnesium anode side to form “dead sulfur”. It is also necessary to ensure that the catalysts are fully exposed without being covered by sulfur species and passivated to effectively enhance the reversibility of Mg‐S batteries. As shown in **Figure** **4**, the double electric layer (EDL) is composed of the diffusion layer and the Helmholtz layer, among which the Helmholtz layer is further divided into the inner Helmholtz plane (IHP) and the outer Helmholtz plane (OHP). In liquid electrolytes, Mg^2+^ is usually solvated by solvent molecules and anions. OHP and IHP respectively represent the closest distances of solvated Mg^2+^ and specifically adsorbed ions.^[^ ^34^ ^]^ The diffusion of polysulfides from the bulk phase through EDL to the surface and the adsorption of the electrolyte solvents hinder the transport of ions and the subsequent continued conversion of sulfur.^[^ ^35^ ^]^ The EDL composition on the surface of the S cathode is reconstructed by adding catalytic sites. Due to the catalytic effect of the catalyst on the desolvation and adsorption of Mg^2+^, the Mg(solvents)_x_^2+^ structure will be attracted to move towards the cathode/electrolyte interface. When the Mg(solvents)_x_^2+^ structure comes into contacting with the surface of the S cathode, Mg^2+^ undergoes rapid desolvation due to the catalytic effect of the catalyst for future conversion reactions. Compared with the blank sample with a significant increase in solvent molecules in IHP, the catalyst restricts a large amount of free Mg^2+^ and polysulfides in the three‐phase interface, further accelerating the subsequent sulfur/sulfide conversion.

## 3 Rapid Sulfur/Polysulfide Conversions with Accelerated Kinetics by Catalysts

Similar to Li‐S batteries, Mg‐S batteries also encounter the problems of poor electrochemical kinetics and soluble polysulfide shuttles, leading to low specific capacity, low cycle life, and low coulombic efficiency.^[^ ^36^ ^]^ In order to obtain a practical high‐performance Mg‐S battery, it seems too much important to understand the reaction kinetics. Among the charge/discharge process of sulfur/sulfide redox reactions, obvious phase transition from solid sulfur to liquid polysulfides and finally to solid magnesium sulfide, exhibiting large reaction barriers. Meanwhile, the phase transformations also result in slow electrochemical kinetics and the accumulation of soluble polysulfide. Therefore, it is necessary to adsorb soluble polysulfide at fixed sites, and then promote the transformation of polysulfide, the catalytic strategy of “adsorption + conversion” can effectively inhibit the polysulfides shuttle, reduce the conversion reaction barrier and enhance the reaction kinetics. In the following, we discuss in detail the strategies for improving the conductivity of cathode, inhibiting soluble polysulfides shuttle, and catalyzing sulfur/polysulfide conversion.

### 3.1 Electric Highway Network Designs for Sulfur Cathode

**Figure 5:**
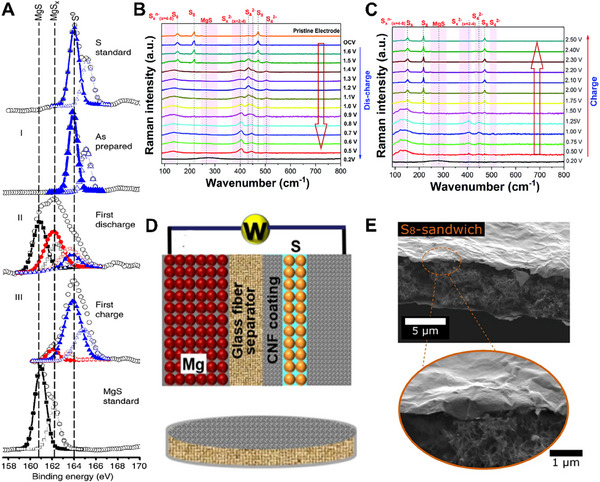
A) XPS S 2p spectra of the cathode as‐prepared (I), after first discharge (II), and after first charge (III) are compared with standard samples of S powder (top) and MgS powder (bottom). Reprinted with permission.^[^ ^38^ ^]^ Copyright 2011, Springer Nature. *Operando* Raman spectra of the S/NC cathode in a Mg‐S battery during B) the discharge and C) the charge. Reproduced with permission.^[61]^ Copyright 2024, The Royal Society of Chemistry. D) Schematic of a Mg‐S battery with the CNF‐S cathode and CNF‐coated separator, and the CNF‐coated separator. Reprinted with permission.^[^ ^42^ ^]^ Copyright 2016, American Chemical Society. E) SEM micrographs of the S_8_‐sandwich film. Reprinted with permission.^[^ ^45^ ^]^ Copyright 2021, Wiley‐VCH GmbH.

In the cathode part, the conductive matrix is an important part for loading insulative sulfur. The reason can be attributed to the poor conductivity, and electron transfer cannot be achieved if all sulfur is used as a cathode. The common solution is to combine the active sulfur materials with various carbon materials such as carbon black, porous carbon, graphene, carbon nanotubes.^[^ ^37^ ^]^ One‐dimensional carbon materials effectively solve the problem of non‐conductivity of sulfur in the research of Mg‐S batteries. Initial attempts to study sulfur cathodes were based on a simple physical mixture of sulfur powder with carbon black or acetylene black. Kim, H. S. et al. directly mixed elemental sulfur, carbon black and a polymeric binder as cathode material to match the designed non‐nucleophilic hydrophilic solution.^[^ ^38^ ^]^ The prepared Mg‐S battery displayed an excellent discharge capacity shows at the first cycle, which confirmed that ordinary 1D carbon material can effectively improve the conductivity of the sulfur cathode to achieve acceptable initial capacity. However, the second discharge capacity of the cathode drops sharply, and then the capacity attenuation is further analyzed. The battery was dismantled after a discharge/charge cycle showed apparent yellow discoloration at the separator, indicating that there is a dissolution of the magnesium polysulfides for the overcharging behaviors of the battery. **Figure** **5A** determined changes in the sulfur oxidation state on the cathode surface during the cycling through XPS. The oxidation state of sulfur in the cathode changes significantly after discharge, and most of sulfur is reduced to a lower oxidation state, which indicates that the binding energy is low. Despite of carbon black, carbon nanotubes and carbon nanofibers, as common one‐dimensional conductive carbon materials, are also commonly used as matrix materials for sulfur cathode.^[^ ^39^ ^]^ In practical fabrication, one‐dimensional materials are easy to stack to form three‐dimensional structure, which can not only promote the conduction of electrons, but also adsorb decomposed polysulfide to a certain extent, providing enough space for volume expansion.

Two‐dimensional graphene and derives are another carbon material to load with sulfur because of their unique characteristics such as high electrical conductivity, large specific surface area, good chemical stability, high charge mobility, excellent electronic and thermal conductivity, and high mechanical strength. For instance, sulfur was dispersed on the reduced graphene oxide (rGO) by chemical precipitation method, which is the first time the development of a Mg‐S battery using a graphene‐sulfur nanocomposite as the cathode.^[^ ^7b^ ^]^ Elemental sulfur material was evenly dispersed across the graphene layer due to (1) its two‐dimensional high surface area, (2) its porous structure, and (3) the presence of various polar oxygen functional groups on the rGO. Vinayan et al. prepared the nitrogen‐doped hybrid nanocomposites with multi‐walled carbon nanotubes (MWCNTs) and graphene as substrates, which was loaded with adjustable amounts of sulfur (0.5‐3 mg cm^−2^) as cathode. The formation of various polysulfide species during the discharging/charging of the Mg‐S battery was investigated by *operando* Raman spectroscopy.^[^ ^40^ ^]^ *Operando* Raman spectroscopy of the Mg‐S battery showed the formation of MgS at the end of discharge from bulk sulfur (S_8_) through a series of long and short chain polysulfides such as S_8_^n−^/S_4_^n−^/S_2_^n−^/MgS (Figure 5B‐C). The main electrochemical reactions in the whole redox process can be summarized as follows: (1) reduction of elemental bulk (S_8_) sulfur to long‐chain polysulfides (S_8_^n−^), (2) reduction of long‐chain polysulfides (S_8_^n−^) to short‐chain polysulfides (e.g. S_4_^2−^/S_2_^2−^), (3) solid‐state transformation of short‐chain polysulfides to MgS. Activated carbon cloth (ACC) has also been receiving attention as a promising carbon host material for Mg‐S batteries.^[^ ^16^ ^]^

Compared with 1D and 2D carbon materials, 3D carbon materials can not only improve the conductivity of sulfur cathode, but also provide more space to accommodate the volume expansion of sulfur and sulfide during the redox reactions. The conductive mesoporous carbon framework (CMK‐3) is a kind of mesoporous carbon material with high order pore height, uniform diameter and large pore volume.^[^ ^17^, ^41^ ^]^ The interconnected pore structure provides a conductive framework for the transfer of electrons and ions, a tight electrical contact for insulating sulfur, and a confinement space for electrochemical reactions. As mentioned earlier, one‐ or two‐dimensional stacks of carbon materials form a three‐dimensional network of conducting carbon. The carbon nanofiber (CNF) can be used as cathode substrate to fill the sulfur‐active material and CNF‐coated separator at the same time (Figure 5D).^[^ ^42^ ^]^ The layer of CNF film was deposited on the separator as the upper collecting layer, which improved the utilization rate of sulfur and the cycle performance of the battery. The prepared cell exhibited the capacity of 1200 mA h g^−1^ at 0.02 C. Alternatively, MXene is another family of 2D layered transition metal carbides or nitrides, which was widely used in Li‐S batteries as sulfur host materials since 2011.^[^ ^43^ ^]^ The representative MXene, Ti_3_C_2_T_x_, has one of the highest electrical conductivity among all solution‐treated nanomaterials.^[^ ^44^ ^]^ Kaland et al. first reported MXenes as a sulfur host material for Mg‐S batteries to improve sulfur utilization and cycle life while reducing inactive weight.^[^ ^45^ ^]^ Generally, they prepared a three‐dimensional sandwich structure of sulfur cathode using MXenes and CNT, which the thinner MXene layer could act as an extremely thin and flexible current collector, the upper layer could act as an interlayer to confine the polysulfide reservoir between the MXene layers, and the carbon nanotubes can complement structures with high surface area for fast dynamics (Figure 5E). MXene and CNTs had a synergistic effect in that CNTs provide a high surface area and prevent the accumulation of MXene, while polar surface groups of MXene reduced polysulfide shuttling and improve coulomb efficiency and capacity retention.

### 3.2 Sulfidophilic Strategies to Confine Polysulfide Shuttling

The diffusion of magnesium polysulfides and deposition of insoluble redox products inevitably hinder the migration of Mg^2+^, affecting the reversibility of sulfur conversions. The reason is that soluble polysulfide is easily formed during the redox process of sulfur cathode. Therefore, it is necessary to design novel sulfur cathode structures to effectively adsorb polysulfide so as to improve the electrochemical performance. The common ways of adsorption of polysulfide can be mainly divided into physical adsorption and chemical adsorption via the sulfidophilic sites or volumetric confinement. Physical adsorption is mainly to use the three‐dimensional structure of the cathode to provide sufficient space and network to inhibit the shuttle of polysulfide, while chemical adsorption is mainly through the chemical bond on the material to play an “attraction” effect on polysulfide, thus adsorbing the polysulfide produced in the reaction.^[^ ^4^, ^42^, ^46^ ^]^

**Figure 6:**
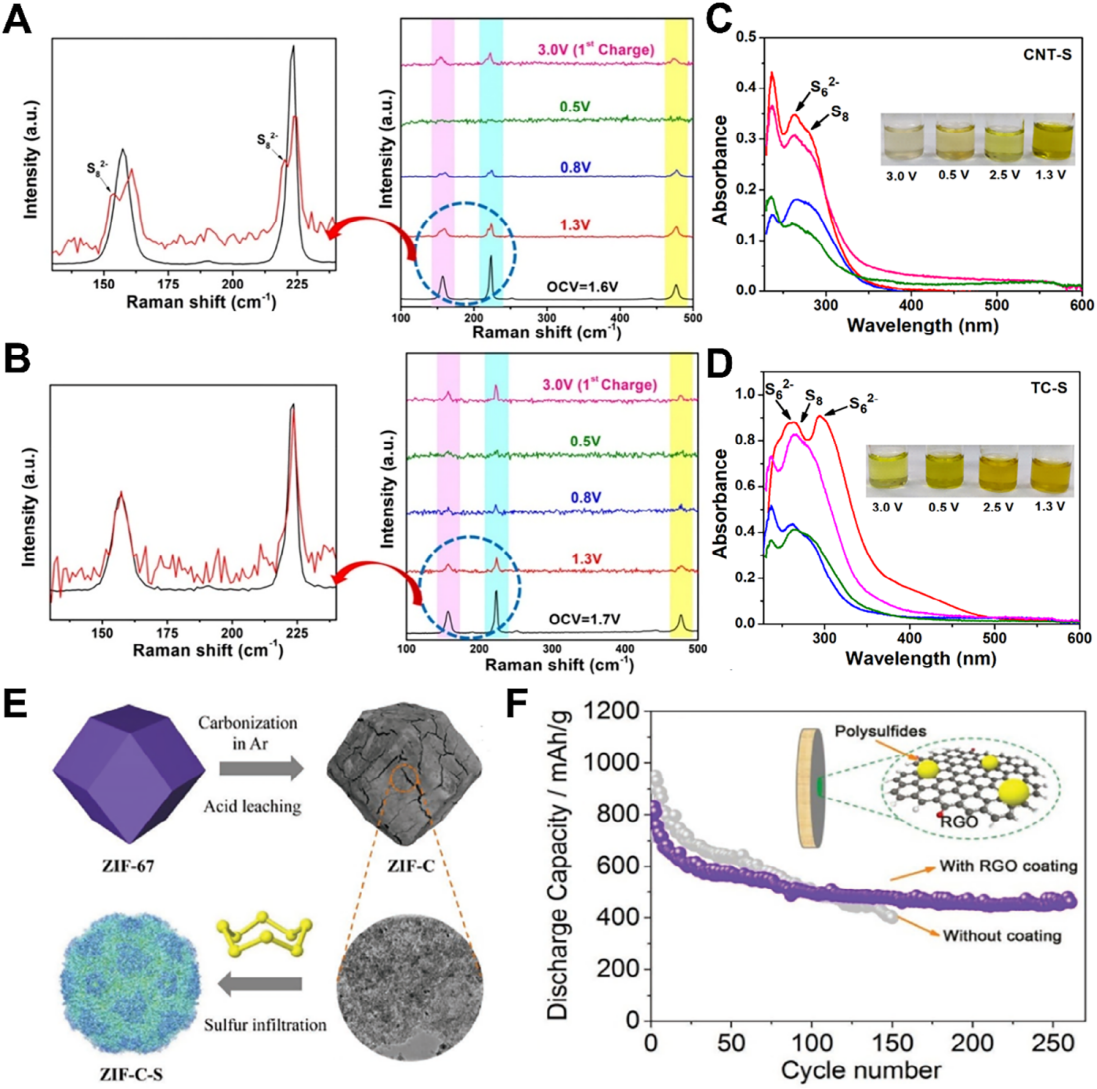
*Operando* Raman spectra of the Mg‐S battery at different discharge voltages using A) CNT‐S and B) TC‐S. Magnified Raman spectra at 1.3 V (red) and OCV (black) for both cathodes are shown in the enlarged image. Comparative UV‐visible spectra of the solutions at different discharge voltages of 1.3 V (red), 0.5 V (green), and charge voltage 2.5 V (pink), 3.0 V (blue) obtained after dilution C) for the CNT‐S cathode and D) for the TC‐S cathode (photographs of solutions obtained upon soaking the separators taken from batteries after different charge/discharge voltage for CNT‐S and TC‐S cathodes are shown in the inset). Reprinted with permission.^[^ ^47^ ^]^ Copyright 2022, American Chemical Society. E) Schematic illustration to prepare ZIF‐C and ZIF‐C‐S. F) Capacity stability comparison between Mg‐S batteries with separator coated by RGO and without separator modification at 0.1 C. Reproduced with permission.^[^ ^37a^ ^]^ Copyright 2018, WILEY‐VCH.

Physical adsorption is mainly attributed to the three‐dimensional network structure formed by carbon matrix to adsorb polysulfide. Bhardwaj et al. used *operando* Raman and post‐cycling *ex‐situ* UV‐vis spectroscopy to investigate magnesium polysulfide confinement in two structurally different porous materials, toray carbon paper (TC) and multi‐walled carbon nanotubes (CNT).^[^ ^47^ ^]^ **Figure** **6A‐B** show the *operando* Raman spectra of Mg‐S batteries employed with CNT‐S and TC‐S cathodes at different discharge voltage depths, respectively. The peaks at 153 and 219 cm^−1^ were due to the presence of high‐order polysulfides, which were confirmed to be limited in the CNT‐S cathode carbon nanotubes. A difference in the color of the solution was observed. In the case of CNT‐S, the low yellowish solution indicated less dissolution of the intermediate polysulfide (Figure 6C‐D). These results together confirmed that the intrinsic structure of carbon nanotubes was more suitable for binding sulfur and polysulfide. Exploring the size diversity model porous electrode/host system will greatly help unlock the complexity of magnesium chemistry, so advanced sulfur hosts should be designed and improved to have strong polysulfide confinement, resulting in high performance Mg‐S batteries. As a unique class of open framework (or porous) materials, metal‐organic frameworks (MOFs) have attracted great interest in recent years due to their ultra‐high surface area and diverse structural topologies.^[^ ^48^ ^]^ In 2018, Zhou et al. proposed a ZIF‐67‐derived carbon framework co‐doped with N and Co atoms as an effective sulfur host to match with bis‐hexamethyldisilazide magnesium (HMDS)_2_Mg based electrolyte with LiTFSI as mediator for highly reversible Mg‐S batteries.^[^ ^37a^ ^]^ Figure 6E shows the schematic diagram of preparation of ZIF‐C and ZIF‐C loaded with sulfur (denoted as ZIF‐C‐S). This hetero‐doped nanoporous carbon had been shown to be effective in trapping soluble polysulfides. They were also trying to combine a variety of strategies to achieve high performance Mg‐S batteries, including the layered porous structure of ZIF‐C, Li salts, and Cl^−^ in addition, the charging mode, and a RGO coated separator (Figure 6F). Consequently, the ZIF‐C‐S had a high S loading capacity of 47%, and its initial discharge capacity was as high as ≈600 mA h g^−1^ 1 C, the reversible capacity remains around ≈400 mA h g^−1^ after 150 – 250 cycles.

**Figure 7:**
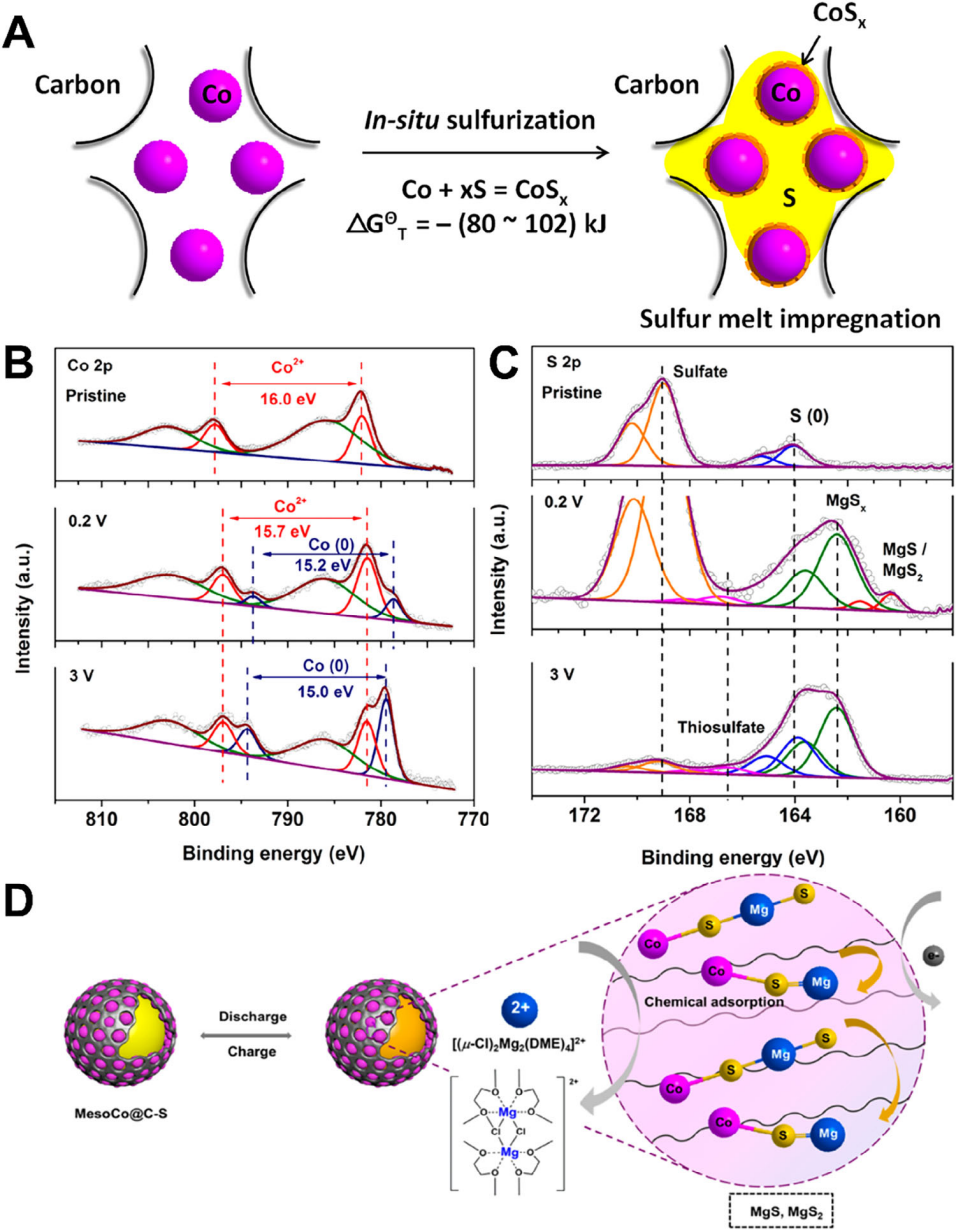
A) Schematics of the surface sulfurization cobalt from the MesoCo@C precursor. *Ex situ* XPS spectra of the MesoCo@C‐S cathode in Mg‐S batteries at different electrochemical states: pristine, discharged to 0.2 V, and charged to 3 V, B) Co 2p and C) S 2p. D) Schematic illustration of the mechanisms for the Mg‐S battery with the MesoCo@C‐S cathode. Reprinted with permission.^[^ ^53^ ^]^ Copyright 2020, American Chemical Society.

In addition to the three‐dimensional structures of physical adsorption of soluble polysulfide, the certain characteristics of the materials shows the merits in carrying out chemical adsorption of polysulfide. For example, defect‐rich MXene material has the characteristics of rich surface functional groups, which can effectively inhibit the “shuttle effect” of polysulfides.^[^ ^49^ ^]^ The hydroxyl group on MXene could be replaced by sulfur and/or sulfide, resulting in strong Ti‐S interaction and effectively inhibiting the “shuttle effect” of polysulfides.^[^ ^50^ ^]^ As a new carbon allotrope, graphylene (GDY) has many interesting properties due to its unique structure. The highly active carbon‐carbon triple bond energy of GDY reacted with short sulfur units and restricts them to internal triangular pores.^[^ ^51^ ^]^ Vanadium nitrogen (VN) hollow nanospheres were selected as sulfur host because of their high chemical absorption characteristics to polysulfides, which could effectively prevent shuttle effect and have better conductivity.^[^ ^52^ ^]^ In addition to the effective chemisorption properties of material for polysulfides, the researchers also focused on more elaborate sulfur cathode designs. In 2020, Sun et al. designed mesoporous matrix (MesoCo@C‐S) as the sulfur cathode. In the process of preparation, carbon confined cobalt sulfide was produced by in situ sulfurization method (**Figure** **7A**).^[^ ^53^ ^]^ Due to the high surface energy of nanosized Co particles, their surface was easy to be oxidized by high temperature sulfur melt to form surface CoS_x_ coating. The sulfurized CoS_x_ species at the surface had inherent electrical conductivity and strong binding energy with polysulfide. Thus, it could achieve surface‐mediated redox reactions by reducing the energy barrier at multiple stages of the S/S^2−^ redox reaction. Both the carbon matrix and the preserved Co core have good electronic conductivity and promote the transfer of charge to sulfide, thus improving the cathode dynamics. *Ex situ* XPS spectra of the MesoCo@C‐S cathode in Mg‐S batteries at different electrochemical states showed, when discharged to 0.2V, Co^2+^ showed a slight deviation at 781.5 eV, which could be considered as the chemisorption between Co and polysulfides. When the battery was charged to 3 V, the Co^2+^ peak splitting to 15.7 eV was still visible from the spectrum, indicating that the residual Co nanoparticles were able to efficiently capture the sulfide chemically (Figure 7B‐C). The end product, MgS/MgS_2_, was detected, which was a good indicator of successful magnesification of sulfur. The Co‐S species on the cathode surface played an important role in trapping magnesium polysulfide by chemical bonding, with the end product Co‐MgS_x_ (*x* = 1, 2) on the surface. Co(0) in bulk material is beneficial to promote cathode dynamics and electron transfer. The mesoporous spheres significantly reduced the volume variation of product formation (MgS, MgS_2_) and inhibited the shuttle of polysulfide to the anode. In the sulfureted poly(acrylonitrile) composite cathode (SPAN), sulfur is chemically bound to the carbon matrix, rather than by physical adsorption. The dominant role is played by vinylogous/phenylogous enolic thioamides, which allows the formation of intramolecular and intermolecular polymer‐S_x_‐polymer chains (2 ≤ *x* ≤ 8) (Figure 7D).

### 3.3 Catalytic Optimization Toward Fast Sulfur/Polysulfide Conversion Kinetics

**Figure 8:**
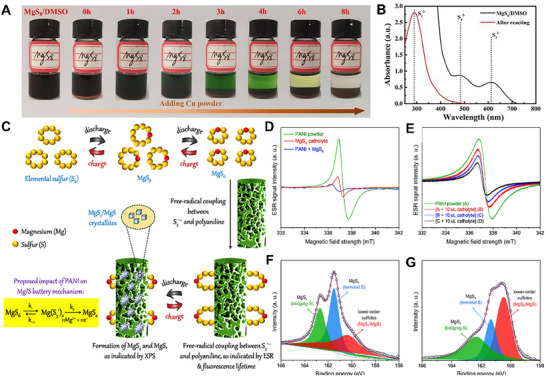
A) Optical photos of Cu powder and MgS_8_/DMSO solution at different reaction periods. B) UV‐spectra of the supernatant before and after the reaction. Reproduced with permission.^[^ ^54^ ^]^ Copyright 2023, Elsevier. C) Schematic diagram of catalytic polysulfide conversion. D) ESR spectra of the individual polyaniline powder, the MgS_x_ catholyte, and their mixture. E) ESR spectra recorded on polyaniline powder during continuous addition of the MgS_x_ catholyte. Deconvoluted S 2p_3/2_ XPS spectra of F) the CC@MgS_x_ cathode and G) the CC@PANI@MgS_x_ cathode after complete discharge. Reprinted with permission.^[^ ^55^ ^]^ Copyright 2022, American Chemical Society.

In order to promote the “solid‐liquid‐solid” phase transition between sulfur and sulfide in the redox reactions, appropriate catalysts need to be proposed to decrease the related electrochemical barriers. Initially, Cu powder can be used as an additive to KB/S cathode to improve the electrochemical performance of Mg‐S batteries, and the performance improvement was somehow proportional to the amount and active sites of Cu powder catalyst.^[^ ^54^ ^]^ The mechanism of the electrochemical reactions of Cu metal on sulfur electrode was further discussed. The results confirmed that the chemical reactions can occur between Cu metal and the intermediate product MgS_8_, and the reaction product Cu_2_S continues to participate in the following electrochemical reactions, finally forming Cu metal and MgS. With the extension of reaction time, the brown‐red solution gradually became clear and transparent (**Figure** **8A**). After the reaction, the characteristic peaks of S_8_^2−^ and S_6_^2−^ in the solution disappeared (Figure 8B). The above phenomenon indicates that chemical reactions can occur between Cu and MgS_8_, indicating the fast catalytic redox process. Combined with XRD, XPS and other characterization methods, the chemical reaction between Cu and MgS_8_ can be inferred as: MgS_8_ + 14Cu→7Cu_2_S + MgS. To improve the dynamics, a new electrode structure was designed using a conducting polymer with a carbon network, which using polyaniline (PANI) as a catalyst (Figure 8C).^[^ ^55^ ^]^ The electrocatalytic activity of PANI in Mg‐S batteries was further studied by electron spin resonance spectroscopy (ESR) spectroscopy and XPS characterization. The researchers proposed that PANI can fix S_3_^•−^ on the cathode to form a chemical adduct through free radical coupling mechanism. This accelerated the electron transfer across the current collector, leading to continuous reduction of S_3_^•−^ to form MgS_2_, and finally to form MgS. It was proved that PANI and S_3_^•−^ species formed reasonable adduction through free radical coupling, which were the characteristics of the end discharge product ESR signal intensity gradually inhibition, peak position movement and line width widening. At CC@PANI@MgS_x_ cathode, the relative peak strength of low‐order polysulfides is higher (Figure 8D–E), indicating that electrochemical conversion of MgS_x_ to end‐discharge products occur to a greater extent. The results of ESR and XPS analysis showed that PANI catalyzed the redox conversion of single anion of polysulfide magnesium to low order polysulfide magnesium by free radical coupling reaction (Figure 8F–G).

**Figure 9:**
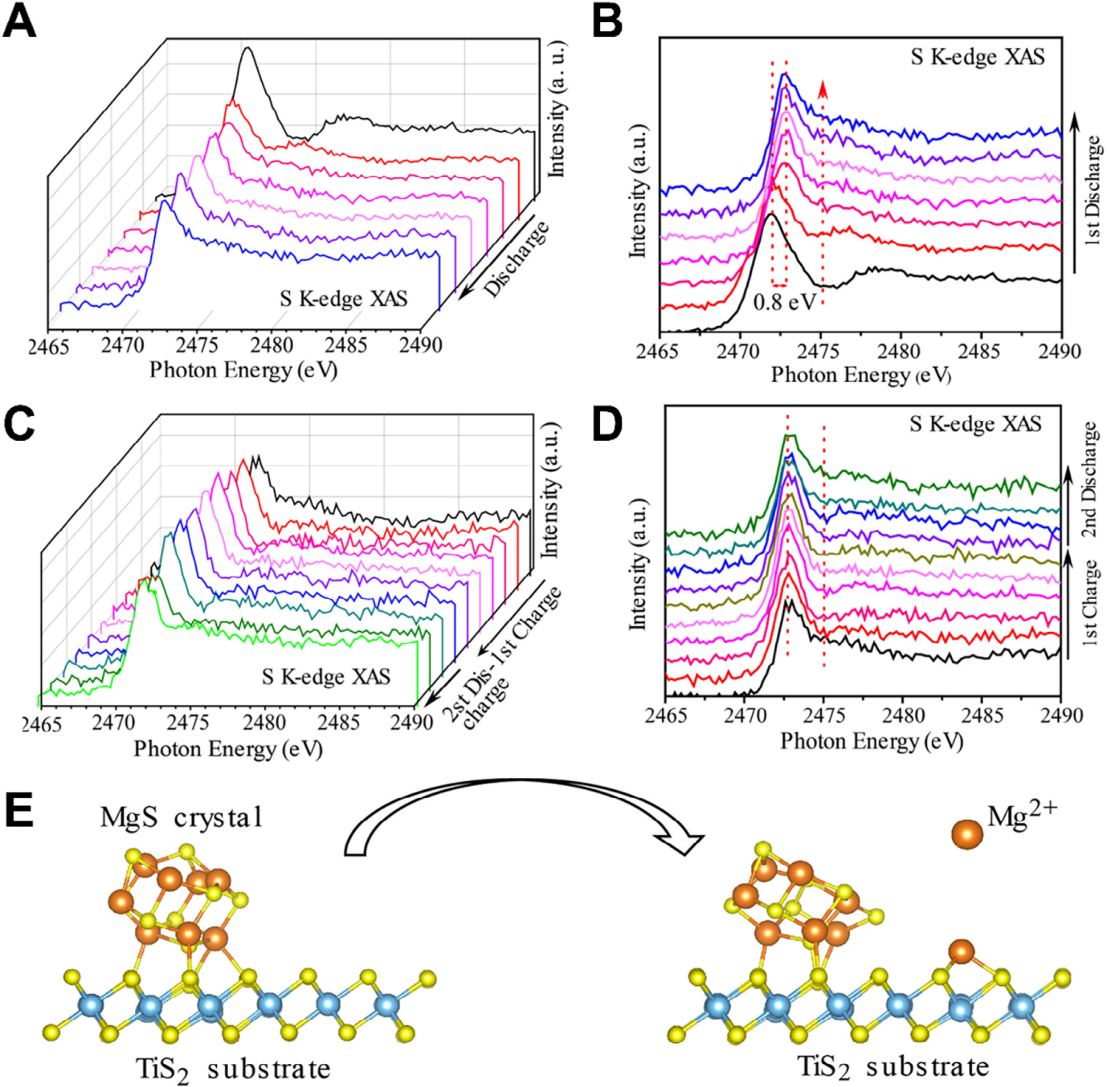
A, B) S K‐edge XAS spectra collected during the first discharging process of the Mg‐S battery at a C/50 rate. C, D) S K‐edge XAS spectra collected during the first charging and second discharging processes of the Mg‐S battery at a C/30 rate. E) Schematic diagram of MgS decomposition on the TiS_2_ surface. Reprinted with permission.^[^ ^20^ ^]^ Copyright 2019, American Chemical Society.

In addition to the introduction of catalysts, it is also an effective means to directly use materials with catalytic capacity as the substrate material of cathode when designing S cathode structure. Nitrogen‐doped mesoporous carbon (NdMC) materials with suitable nitrogen content and surface groups can be used as catalytic hosts to enhance the ability of magnesium polysulfide and electrons to enter redox active sites in Mg‐S batteries.^[^ ^56^ ^]^ Xu et al. used synchrotron X‐ray absorption spectroscopy (XAS) to reveal the reaction pathway of Mg‐S batteries, which could provide detailed chemical environment and oxidation state information for specific elements in complex systems.^[^ ^20^ ^]^ In addition, the *in‐situ* XAS method could study real‐time changes in the material's electronic structure under near‐real battery operating conditions. In the first discharge process, the transformation of S could be divided into three stages: the formation of high‐order MgS_x_ (MgS_8_, MgS_4_) with the rapid reaction rate, the reduction of MgS_4_ to Mg_3_S_8_, and the slow further reduction of Mg_3_S_8_ to MgS. However, Mg_3_S_8_ and MgS were electrochemical inert and were difficult to reverse back to higher order polysulfides or S based on *in‐situ* XAS results (**Figure** **9A–D**). The interaction of TiS_2_ with Mg is beneficial to the decomposition of Mg‐S bonds (Figure 9E) and can activate unreacted MgS and Mg_3_S_8_ species, improving the performance of Mg‐S batteries.

**Figure 10:**
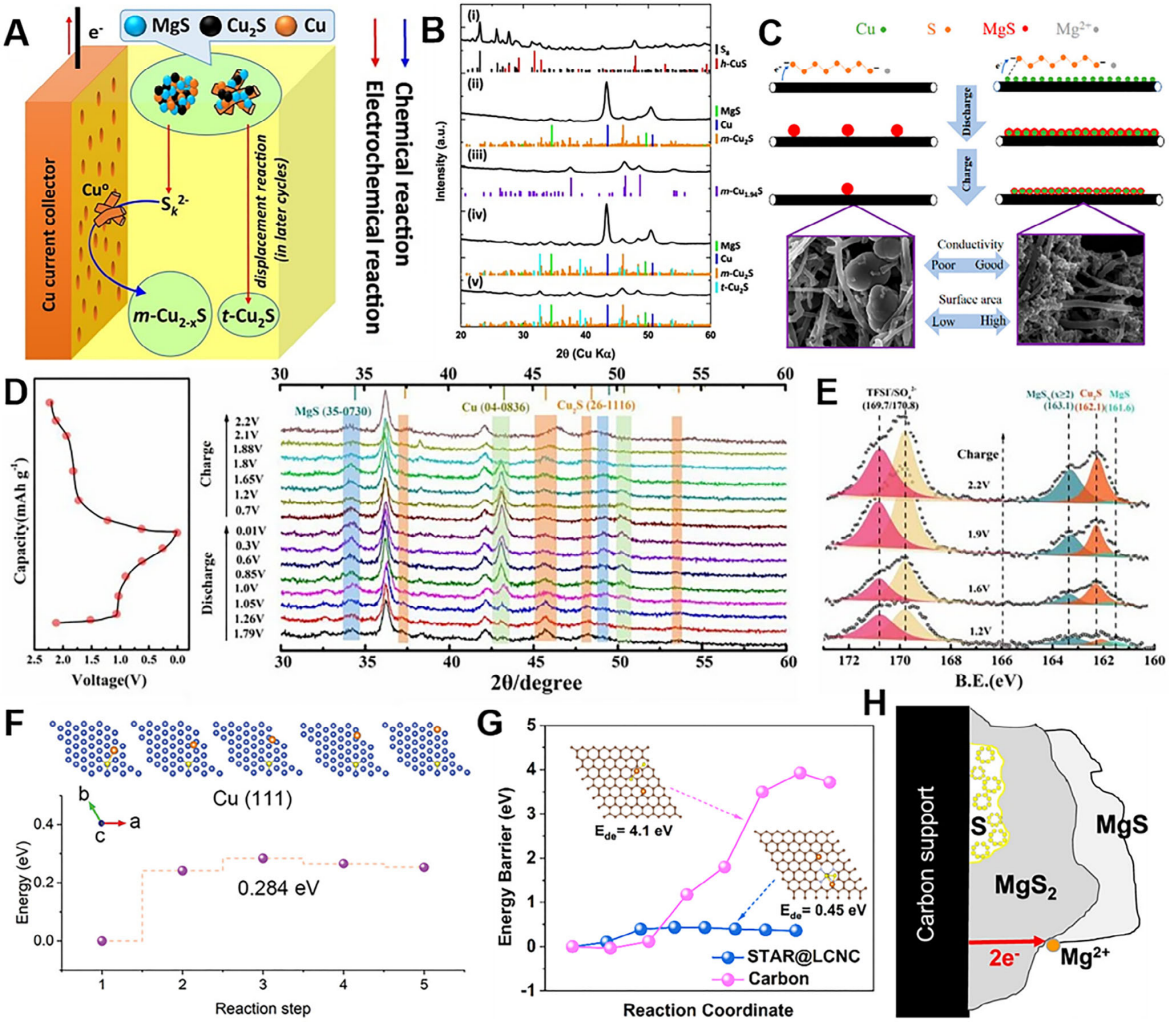
A) Schematic diagrams describing the electrode process occurring on the sulfur cathode employing a Cu current collector during charge. B) XRD patterns of the pristine and cycled sulfur cathodes employing Cu current collector with stick patterns for the reference phases. Reproduced with permission.^[^ ^57^ ^]^ Copyright 2019, Elsevier. C) The charge and discharge process of Mg‐S batteries under the action of Cu nanoparticle additives. Reprinted with permission.^[^ ^58^ ^]^ Copyright 2019, American Chemical Society. D) The XRD patterns of Cu‐KB/S cathode at different steps of dis‐ and charge process. E) the XPS spectra of Cu‐KB/S cathode at different steps of charge process. Reproduced with permission.^[^ ^54^ ^]^ Copyright 2023, Elsevier. F) The simulated pathways along the decomposition process and corresponding energy profiles of MgS on Cu (111) surfaces. Reproduced with permission.^[^ ^30^ ^]^ Copyright 2024, WILEY‐VCH. G) Comparison of decomposition energy barriers of Mg_2_S_2_ cluster on STAR@LCNC and carbon, respectively. Reproduced with permission.^[^ ^22^ ^]^ Copyright 2024, The Royal Society of Chemistry. H) Mg^2+^ ions from the electrolyte react with the MgS_2_ surface to form a MgS deposit. The conduction of electron polarons in MgS_2_ provides the charge for the redox process. Reproduced with permission.^[^ ^59^ ^]^ Copyright 2023, American Chemical Society.

The oxidation of MgS is much more important in the reversibility. During the discharge process of Mg‐S batteries, Mg and S theoretically react to form MgS, and the Gibbs free energy for generating MgS is negative, which proves that this reaction is thermodynamically spontaneous. On the contrary, the reverse reaction of MgS converting to Mg and S is thermodynamically non‐spontaneous. Therefore, the oxidation of MgS is usually a key step in the reversible charge and discharge of magnesium‐sulfur batteries. Generally, the activation of MgS can be realized by the screening of current collector. the Copper, as a conventional current collector material, can chemically react with polysulfides generated from sulfur or MgS with Mg^2+^.^[^ ^57^ ^]^ During the charge, metallic copper reacts with MgS to form Cu_2_S, while trace amounts of Cu^+^ may be extracted from the densely packed Cu_2_S or Cu particles, creating subtle cationic defects (**Figure** **10A**). These defects subsequently incorporate into the MgS matrix, thereby enhancing the reaction kinetics. Consequently, the extraction of Mg^2+^ from MgS is significantly facilitated in the presence of Cu^+^. The formation of Cu_2_S can be attributed to a displacement reaction involving nanoscale MgS in intimate contact with Cu metal, exhibiting remarkable reversibility as it undergoes cyclic conversion between MgS and metallic Cu (Figure 10B). Copper nanoparticles grown on carbon nanofibers can also play a similar role as additives for the cathode of Mg/S batteries, similar to copper current collectors (Figure 10C).^[^ ^58^ ^]^ The *ex‐situ* XRD and XPS results in Figure 10D,E indicate that the introduction of copper powder fundamentally changes the reaction mechanism of the sulfur cathode.^[^ ^54^ ^]^ At the initial stage of the discharge process, a chemical reaction can occur between Cu and the intermediate product MgS_8_. The reaction products of Cu_2_S continue to participate in the electrochemical reduction reaction to regenerate copper, and they can be re‐oxidized to Cu_2_S during the charge process. Compared with the traditional Mg‐S batteries, the redox couple in the cathode are converted from S/S^2−^ to Cu_2_S/Cu^0^, which improves the cycling stability of the battery. Zou et al. investigated the intrinsic mechanism of Cu in reducing the activation energy of MgS with DFT calculations, proposed that the mechanism involves the dissociation of one Mg and one S from MgS, which constitutes the rate‐limiting step during the charge process.^[^ ^30^ ^]^ As illustrated in Figure 10F, MgS exhibits the lowest decomposition energy barrier on the Cu (111) surface. The Mg‐S bond length adsorbed on the Cu (111) surface is longer, which suggested the molecule is more strongly activated by electron redistribution, weakening the Mg‐S bond strength and promoting the decomposition of MgS. In addition to copper, introducing a single atomic metal catalyst at the cathode/electrolyte interface can also effectively reduce the decomposition barrier of MgS (Figure 10G). The energy that needs to be overcome to move a Mg atom away from Mg_2_S_2_ is reduced by nearly ten times under the action of single atomic Zn.^[^ ^22^ ^]^ Through further first‐principles calculations and ab initio molecular dynamics, it was found that MgS_2_ can maintain electronic conduction through the mechanism of double electron polaron species migration.^[^ ^59^ ^]^ Therefore, rechargeable Mg‐S batteries can be developed if it can be ensured that battery discharge does not promote the oxidation process beyond the formation of MgS_2_ (Figure 10H).

In sulfur cathodes, even the different catalysts selected need to be fixed on some carbon material, so the choice of carbon material and catalyst is almost equally important. Although ordinary carbon tubes can provide electrical conductivity, it is difficult to inhibit the shuttle of polysulfides, while three‐dimensional structures such as MOF and MXene can effectively inhibit the shuttle of polysulfides and provide space for volume expansion. Moreover, nitrogen‐containing carbon matrix also has a certain promotion effect on the transformation of polysulfides due to the presence of C‐N bonds. Metal sulfides and metal oxides are common catalysts, but their catalytic activity is also different due to the influence of different metal elements and particle size. The metal single atom catalyst has the highest catalytic activity in theory, but it also faces the problem of easy agglomeration.

## 4 Conclusions and Prospects

**Figure 11:**
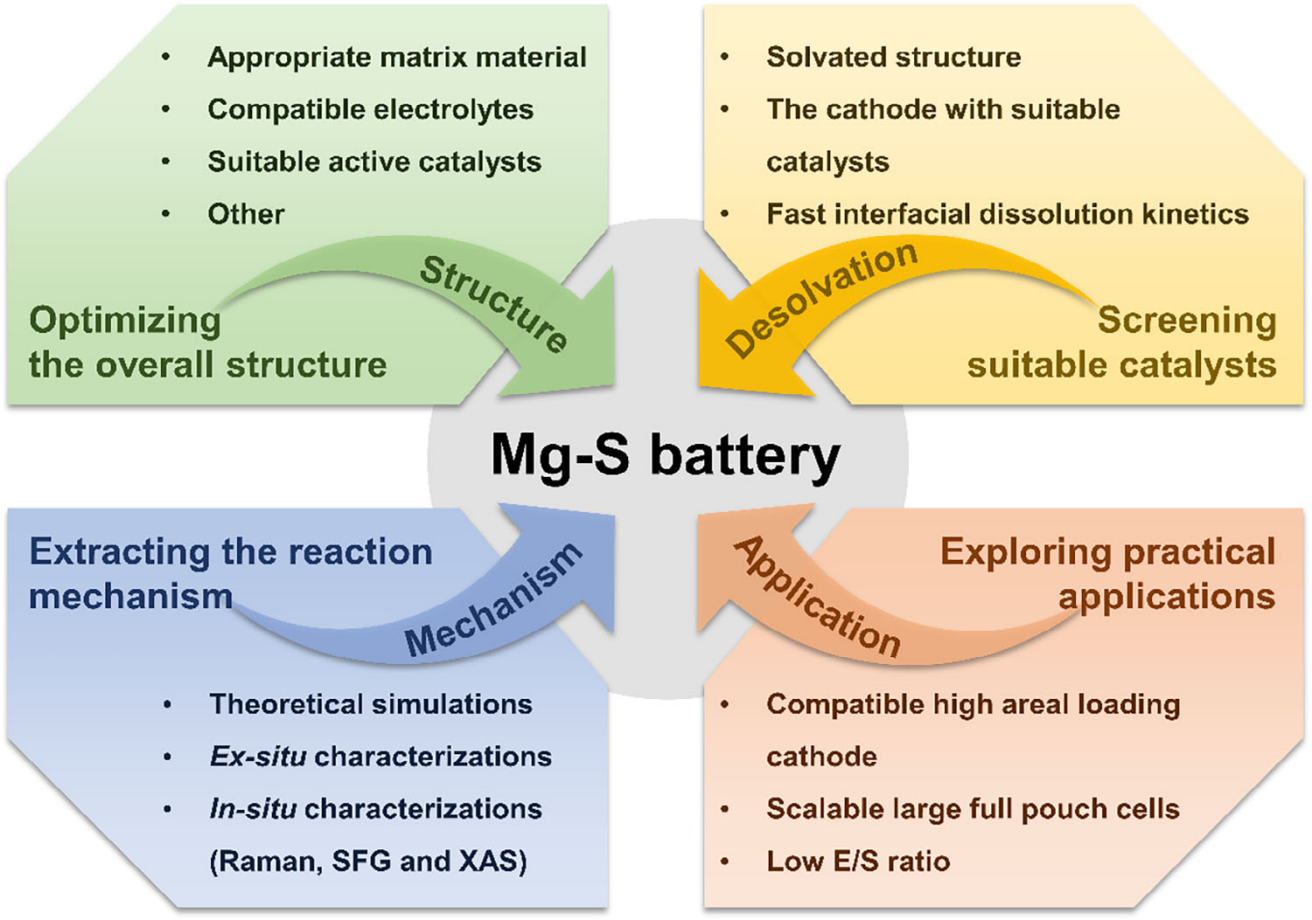
Summary and prospects of desolvation and conversion in Mg‐S batteries for future development.

In summary, sulfur is a typical conversion‐based cathode material in magnesium battery system. Although Mg‐S battery has the advantages of high energy density, low cost and high safety, there are huge reaction barriers in the process of desolvation and sulfur/sulfide conversion. By the help of catalysis, the following ones are systematically analyzed: 1) the specific working mechanism of the Mg‐S battery and the necessity and importance of the desolvation process; 2) the problems and solutions of diffusion/desolvation Mg^2+^ migration barrier at sulfur/electrolyte interface; 3) the current development and design of S cathodes in Mg‐S batteries. The specific working principle of S cathode in Mg‐S battery has not been analyzed and understood, and the improvement of battery performance. In order to obtain better performance of sulfur cathode, more in‐depth analysis of the working mechanism of Mg‐S battery, as soon as possible to obtain stable performance and practical Mg‐S battery, further improvement can be made from the following aspects (**Figure** **11**):

1. *Optimizing the Overall Structure of the Mg‐S Battery from Cathode, Anode to Compatible Electrolytes*: The structure and type of the matrix material have a great influence on the performance of the S cathode. The appropriate matrix material can not only provide a continuous and rapid conductive network to meet its conductivity needs, but also provide enough space and adaptability for the volume change of sulfur to inhibit its fall off failure, and the porous morphology can also realize the physical adsorption effect to slow the shuttle effect of polysulfide intermediates, so that the magnesium sulfur battery can work steadily. But this strategy cannot reduce the high energy barrier in the process of sulfur conversion, resulting in slow kinetics and fast decay, the introduction of polar element doping or highly active catalysts in the construction of cathode matrix materials can effectively adsorb polysulfides and accelerate their conversion. Therefore, the introduction of suitable active catalysts such as transition metal oxides, sulfides, defect rich catalysts (DRCs) and single atom catalysts (SACs) is expected to significantly improve the performance of Mg‐S batteries, improve the reaction kinetics of polysulfide conversion and promote the transport of magnesium ions. For the magnesium anode, design a modified layer that can isolate the contact with the electrolyte and enable rapid ion conduction, or explore new magnesium alloy materials to reduce the possibility of the formation of a passivation layer on the surface of the magnesium foil, make the deposition of magnesium ions more uniform, and extend the service life of the battery. The optimization of the compatible electrolyte is of utmost importance. Developing electrolytes with high ionic conductivity and excellent chemical stability, enabling them to efficiently conduct magnesium ions between the cathode and anode while suppressing the occurrence of side reactions. Through the collaborative optimization of various parts, Mg‐S batteries are expected to achieve significant breakthroughs in key indicators such as energy density, cycle life, and safety.
2. *Screening Suitable Catalysts to Fasten Interfacial Desolvation and Diffusion at Electrode/Electrolyte Interface*: At present, the magnesium battery system lacks stable and widely used commercial electrolyte, and the electrolyte with various complex components is not conducive to the study of solvated structure, and it is easy to form a solvated structure with large ionic radius, which hinders its dissociation and transport at the electrode/electrolyte interface, and Mg^2+^ enter the cathode to participate in the reaction. High performance can only be achieved if a well‐designed cathode allows suitable characteristics such as rapid dissolution and diffusion of the Mg^2+^ interface. Therefore, it is necessary to optimize the cathode, introduce a suitable catalyst at electrode/electrolyte interface, which can effectively reduce the activation energy of the desolvation and sulfur/sulfide conversion reaction, to produce the fast interfacial dissolution kinetics to release more Mg^2+^ and to accelerate the diffusion of free Mg^2+^ into the interior of the cathode for a fast reaction, thus greatly improving the charge and discharge rate of the battery. In the future, research similar to the activation of Li_2_S cathodes will also focus on directly activating MgS with catalysts. Every effort will be made to explore how Mg^2+^ migration under the catalyst and will adopt suitable operando methods to trace the interaction behaviors. With the continuous breakthrough of this technology, Mg‐S batteries are expected to maintain stable performance under high rate charging and discharging conditions. This will open up broad application prospects for Mg‐S batteries in areas such as power tools, emergency power supplies, and other areas with high requirements for charge and discharge speed, and further promote the innovation of energy storage technology.
3. *Extracting the reaction mechanism from combination of theoretical simulations with ex/in‐situ characterizations*: At present, the specific working principle and intermediate state of Mg‐S batteries have not been determined, so more testing means and methods are used to further analyze the working mechanism of Mg‐S batteries. For example, time‐of‐flight secondary ion mass spectrometer (TOF‐SIMS) is used to observe the state of S cathode before and after the charging/discharging cycle, in situ characterization (such as Raman, XRD, SFG and XAS) should also be further introduced to trace the evolution of desolvation and sulfur related substances. Based on a large number of optimization strategies, theoretical simulation is a useful tool, which can significantly simplify the experimental steps and greatly save the experimental cost, such as binding energy, diffusion energy, and Gibb's free energy of each reaction step, and further understand the reaction mechanism of Mg‐S batteries. When the theoretical simulation and *in‐situ* characterization are closely combined, we are expected to reveal the reaction mystery of Mg‐S batteries in a comprehensive and deep way. This will not only promote the Mg‐S batteries to achieve a qualitative leap in performance, such as improving energy density and extending cycle life, but also lay a solid foundation for its large‐scale application in key areas such as electric vehicles and smart grids, and effectively promote the vigorous development of energy storage technology in a more efficient, environmentally friendly and sustainable direction.
4. *Modulating Compatible High Areal Loading Cathode or Scalable Large Full Pouch Cells for Practical Applications*: At present, the research on the design of sulfur cathode in Mg‐S battery system is still in the initial stage. From a practical point of view, increasing the areal loading of sulfur cathodes is one way to obtain high surface capacity to meet commercial needs. However, high areal loading sulfur cathode is bound to face more problems, such as the decrease of conductivity of electrode materials and the intensification of shuttle effect of polysulfide, which often make some strategies difficult to achieve the desired effect. Therefore, it is necessary to take effective measures to increase the sulfur loading of the cathode and optimize the electrochemical performance at low E/S ratio. We can try to develop a new type of sulfur composite electrode material to enhance the stability and reactivity of the electrode by carefully designing the structure and composition of the material. In order to achieve the goal of high energy density, a feasible experiment is to propose a large‐area pouch battery using low electrolytes. Under the premise of high sulfur loading, due to the consumer demand for fast charge and long life in our daily life, fast charge technology and long life should also receive more attention. Given this fact, the use of highly conductive matrix materials combined with highly efficient kinetic promoters, such as nitrogen‐doped carbon network‐supported active SACs, which can offer more potential than ordinary adsorption and catalytic materials, improve the fast charging performance of the battery, inhibit the shuttle of polysulfides effectively, and extend the cycle life of the battery. Once a breakthrough is made in these key technologies, Mg‐S batteries will show great application value in electric vehicles, large‐scale energy storage, and other fields, and effectively promote the energy storage industry to take a big step forward in the direction of efficiency, green, and sustainable.

## Conflict of Interest

The authors declare no conflict of interest.

## Author Contributions

Q.G. contributed to the conceptualization of this review, design and drawing of figures, and writing of the original draft, J.Z., Y.Z., X.C., J.D., and L.J. contributed to the editing and reviewing of the draft, H.L. and J.W. contributed to conceptualization, editing, writing, reviewing, and supervising. All authors discussed and commented on the manuscript.

## Data Availability

No primary research results, software or code have been included and no new data were generated or analysed as part of this review.
